# Metastatic lung adenocarcinoma with *BRCA2* mutation and longstanding disease control on olaparib, developing triple negative breast adenocarcinoma with additional *BRCA2* reversion mutation: a case report

**DOI:** 10.1186/s13256-023-04139-x

**Published:** 2023-09-25

**Authors:** Auro del Giglio, Beatriz da Costa Aguiar Alves, André Márcio Murad, Fernando Luiz Affonso Fonseca

**Affiliations:** 1 Departamento de Oncologia e Hematologia, Centro Universitário FMABC, Av. Príncipe de Gales, 821, Santo André, SP 09060-650 Brazil; 2 Laboratório de Análises Clínicas, Centro Universitário FMABC, Av. Príncipe de Gales, 821, Santo André, SP 09060-650 Brazil; 3 CENANTRON-PERSONAL-Precision Oncology, R. Padre Rolim, 120, Belo Horizonte, Minas Gerais 30130-090 Brazil; 4 Instituto de Ciências Farmacêuticas, Universidade Federal de São Paulo (UNIFESP), Rua Professor Artur Riedel, 275, 09972-270 Diadema, SP Brazil

**Keywords:** BRCA2, Reversion mutation, Therapy resistance

## Abstract

**Background:**

The *BRCA2* gene is a well-known tumor suppressor gene implicated in breast and ovarian cancers. *BRCA1/2* mutations can be sensitive to poly ADP-ribose polymerase (PARP) inhibitors such as olaparib. However, some of these patients develop resistance to this treatment and an essential factor contributing to acquired insensitivity is the occurrence of reversion mutations in the *BRCA1/2* genes.

**Case presentation:**

We report the case of a 65-year-old Brazilian female patient who had previously been diagnosed with metastatic lung carcinoma carrying a *BRCA2* mutation that had extended to the central nervous system. Following disease progression, olaparib was administered, resulting in a stabilizing effect on her condition for ~ 30 months. During a routine follow-up, a new triple-negative breast tumor was found. Genetic testing revealed the presence of two distinct *BRCA2* gene mutations in the breast tumor. The original mutation (p.Val220Ilefs4) led to a frameshift, culminating in the production of a truncated and non-functional BRCA2 protein; the second mutation, K437fs22, rectified the reading frame of exon 11. Consequently, Rad51 could properly bind to BRCA2—an essential protein crucial for DNA repair. This restoration resulted in a functional BRCA2 protein, effectively elucidating the clinical resistance observed in the new breast tumor in this case.

**Conclusions:**

This case report highlights the clinical significance of comprehensive next-generation sequencing analyses for lung adenocarcinomas, both at diagnosis and upon progression. Such analyses enable informed decisions regarding targeted therapies and facilitate a deeper comprehension of resistance mechanisms.

## Background

Lung adenocarcinoma accounts for the highest number of cancer-related deaths worldwide, constituting 18% of them [1]. Among all lung cancer cases, adenocarcinoma makes up ~ 40% [2], and around 60% of adenocarcinomas carry at least one mutation [3]. The most prevalent mutations in patients with lung adenocarcinoma occur in the *EGFR*, *KRAS*, and *ALK* genes [3]. The significance of investigating these mutations lies in the availability of targeted therapies for some of them. These drugs have the potential to alter the natural course of the disease for responsive patients, leading to substantial enhancements in their survival and quality of life [4].

*BRCA1/2* germinative mutations are the most common cause of hereditary breast and ovarian carcinomas, but they can also lead to other types of cancer, such as pancreatic and prostatic carcinomas [5]. Approximately 1% of lung adenocarcinomas can harbor *BRCA1/2* mutations [6], which can be sensitive to poly ADP-ribose polymerase (PARP) inhibitors such as olaparib [7]. However, some of these patients develop resistance to this treatment, and an essential factor contributing to acquired insensitivity is the occurrence of reversion mutations in the *BRCA1* or *BRCA2* genes [8]. A reversion mutation is a genetic alteration that reinstates the functionality of a gene previously mutated [9].

We are reporting a case of a patient with a germline mutation in *BRCA2* and a diagnosis of metastatic lung adenocarcinoma with long-standing disease control on olaparib. The patient developed a new triple-negative breast carcinoma while receiving this PARP inhibitor. Interestingly, next-generation sequencing (NGS) of the breast tumor revealed an additional *BRCA2* mutation.

## Case presentation

We present a case involving a 65-year-old Brazilian woman with a history of smoking and a family background of breast cancer. Her mother passed away at the age of 61 years due to breast cancer, and her maternal aunt also succumbed to breast cancer at the age of 51 years. This patient was diagnosed in August 2019 with lung PDL1 negative adenocarcinoma metastatic to the central nervous system (CNS) with three lesions (right frontal and left occipital and parietal), the largest of them 1 cm in diameter. She subsequently underwent stereotactic body radiation therapy (SBRT) of the three CNS lesions followed by a four-cycle regimen of chemotherapy involving pembrolizumab, carboplatin, and pemetrexed. This was followed by maintenance therapy consisting of pembrolizumab and pemetrexed.

She additionally underwent radiation therapy with a dose of 3000 cGy delivered in five fractions targeting the residual mass in the right upper lung. This approach aimed to enhance the effectiveness of immunotherapy. Maintenance therapy was continued until August 2020. Unfortunately, at this point, disease progression was observed in the central nervous system (CNS), characterized by the emergence of two new cerebellar lesions. In response, she underwent surgery to address the larger of the two lesions and subsequently received stereotactic body radiation therapy (SBRT) for the other lesion. The pathological examination of the cerebellar lesion revealed positivity for thyroid transcription factor-1 (TTF-1), cytokeratin 5, and Napsin, which was consistent with brain metastasis originating from her pulmonary adenocarcinoma.

We had not yet obtained the NGS study of the cerebellar lesion. Since the FoundationOne test of the lung lesion revealed mutations in both the *BRCA2* and *ATM* genes (but not in *EGFR* and *ALK*, as presented in Table 1), we initiated her treatment with olaparib in November 2020. The initial dosage was three tablets of 150 mg per day, which was later reduced to two tablets daily due to severe fatigue. By February 2023, positron emission tomography/computed tomography (PET/CT) scan revealed a new lesion in her right breast. Despite her possession of a germline BRCA mutation (as detailed in Table 1), we did not pursue breast screening due to the metastatic nature of the lung carcinoma.

**Table 1:** Foundation One results from the primary lung adenocarcinoma, primary breast adenocarcinoma, and germinative panel for cancer predisposition

NGS panel	Results
Invitae Multi-Cancer Panel	• BRCA2c.658_659del (p.Val220Ilefs*4) heterozygous—pathogenic • NTHL1c.550-1G > A (splice acceptor) heterozygous—likely pathogenic
FoundationOne lung	• ATM-L2417P • BRCA2-V220fs*4 • STK11-G47fs*4 • KRAS-G12C • Tumor mutational burden (TMB)—Intermediate (11 Muts/Mb) • Microsatellite status (MS)—stable
FoundationOne breast	• BRCA2-A2-V220fs*4 • K437fs*22 • CBFB—loss exons 1–3 • ETV6—rearrangement intron 5 • TP53—loss exons 2–11

**Fig. 1:**
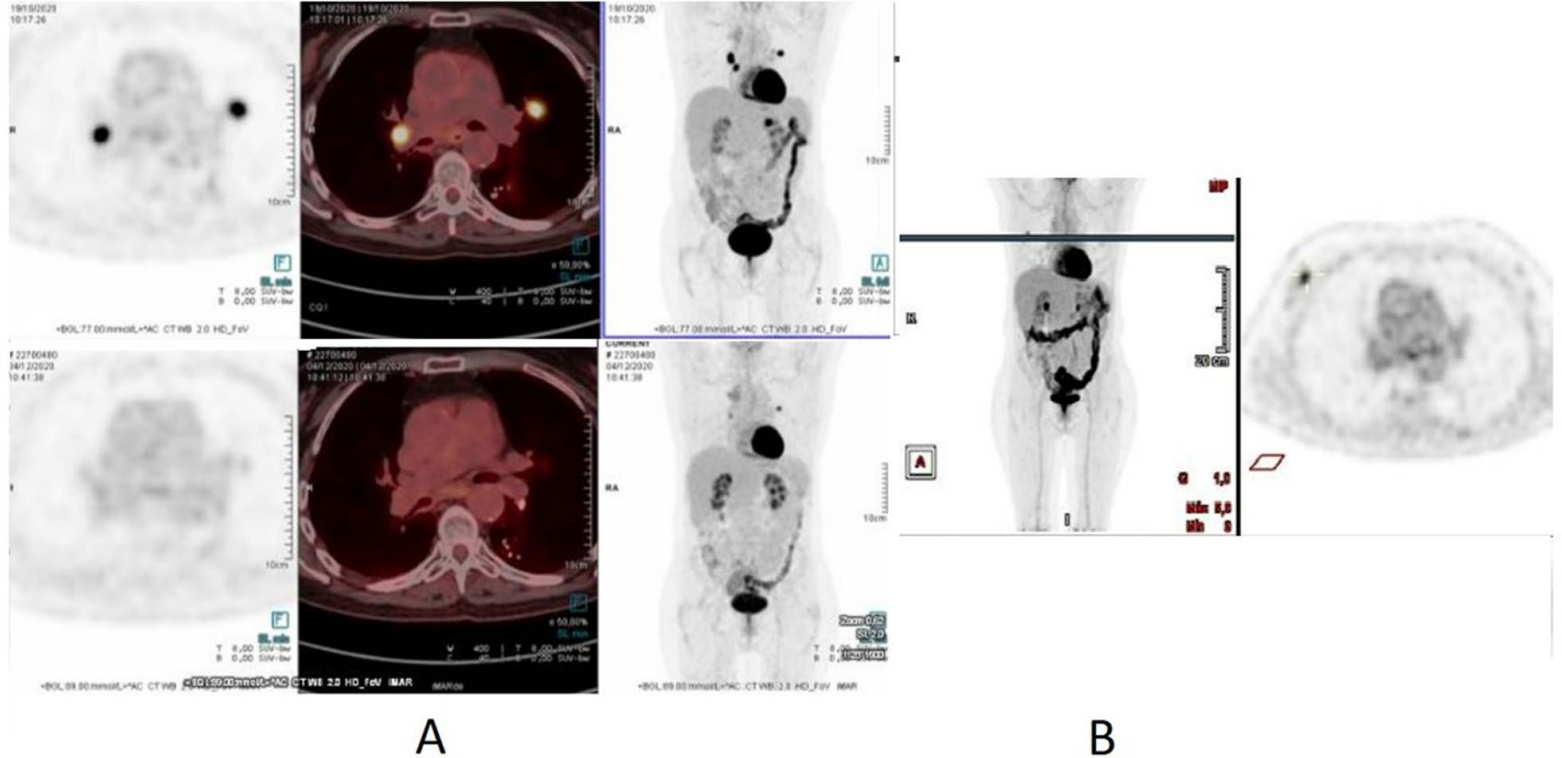
**A** Upper three images: Fluorodeoxyglucose Positron Emission Tomography/Computed Tomography (FDG-PET/CT) scan results before starting olaparib in October 2020. Lower three images of December 2020 after 1 month of olaparib showing a partial response in the mediastinal and lung nodules. **B** FDG PET/CT scan from February 2023 at the level of the right breast showing the appearance of a new hypermetabolic nodule in the right breast and no activity in previous lung and mediastinal nodules

A biopsy of the breast lesion revealed a triple-negative invasive breast adenocarcinoma. Subsequently, she underwent a quadrantectomy, and the pathological report unveiled a 14 mm triple-negative breast carcinoma. Additionally, one sentinel lymph node displayed a 3 mm focus of carcinoma (Fig. 1). Given the overall stability observed in the PET scan results, the decision was made to continue olaparib treatment without interruption. The FoundationOne test results for the breast tumor are also presented in Table 1. In addition to her ongoing treatment, she also received adjuvant radiation therapy targeted at the right breast.

**Fig. 2:**
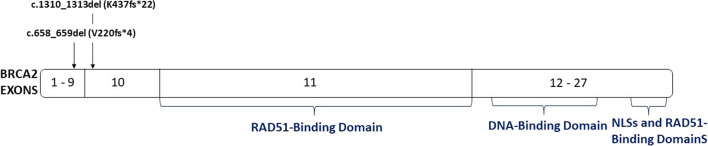
Graphical representation of *BRCA2* gene structure and localization of p.Val220Ilefs*4 and K437fs*22 mutations (exons 8 and 10, respectively) and domains

We analyzed the impact of both mutations using the predictProtein software (freely available at https://predictprotein.org); our analysis revealed that the original *BRCA2* mutation, c.658_659del (p.Val220Ilefs4), results in a frameshift, which in turn leads to the truncation and inactivation of the BRCA2 protein. Conversely, the second mutation, c.1310_1313del (K437fs22), restores the reading frame, initiating from the beginning of exon 10. Notably, exon 10 encompasses a DNA binding domain [10]. Exon 11, in turn, encompasses eight crucial functional domains referred to as BRC repeats, which directly interact with the recombinase RAD51 [10] (Fig. 2). In effect, this second mutation reinstates the functionality of *BRCA2*, which had been disrupted by the initial mutation.

## Discussion and conclusions

This case highlights several intriguing aspects. The initial observation centers around the remarkable extended disease control achieved over a span of 30 months in this patient with olaparib, targeting her metastatic lung adenocarcinoma harboring a *BRCA2* mutation. In existing literature, we encounter instances of transient responses in lung adenocarcinomas with *BRCA2* mutations, ranging from 13 months [11] to 6 months [7]. This case exemplifies why individuals with lung adenocarcinoma, regardless of their smoking history, should ideally undergo comprehensive next-generation sequencing (NGS) to detect potentially rare yet actionable mutations, as underscored by the findings in this patient’s case.

Another interesting observation is that resistance to olaparib appeared in the breast tumor of this patient through a reversion mutation. Reversion mutations are characterized as secondary mutations, often manifesting as small deletions, within a mutant gene. These mutations work to restore the reading frame of the gene, thereby giving rise to a partially functional protein [8]. In this specific case, the original *BRCA2* mutation led to a frameshift, culminating in the production of a truncated and nonfunctional BRCA2 protein. Subsequently, the occurrence of a second mutation reinstated the *BRCA2* reading frame, thus enabling the functionality of exon 11 within this gene. Indeed, exon 11 of *BRCA2* serves as the site where Rad51 binding takes place [8]. Thanks to the presence of this second mutation, the functionality of *BRCA2*, which had been compromised by the original mutation, was reinstated. Consequently, this mutation falls into the category of reversion mutations.

The reason behind the occurrence of this mutation in breast tissue, leading to the emergence of a new primary triple-negative breast tumor, rather than manifesting in one of the previously identified lung primary or metastatic sites, resulting in disease progression at any of those locations, remains elusive. Notably, Darabi *et al*. and Yonina *et al*. [12, 13] also presented findings indicating a higher occurrence of reversion mutations in breast and ovarian carcinomas compared with other tumor histologies. Whether this observation can be attributed to the elevated frequency of BRCA mutations in breast and ovarian cancers, or if there exists a tissue-specific context that facilitates a higher likelihood of reversion mutations, remains to be clarified. Of particular interest is the fact that the use of platinum salts, as seen in the treatment of this patient, has been linked to the occurrence of reversion mutations [13].

We interpreted the emergence of the new triple-negative breast tumor as the sole locus of disease that had become unmanageable. Consequently, we maintained the administration of olaparib, concurrently implementing surgery and radiation therapy to address the breast tumor. We chose not to introduce adjuvant chemotherapy, as we believed it had the potential to jeopardize the intricate task of managing the metastatic lung cancer, which had been effectively controlled using olaparib. Combining chemotherapy with a PARP inhibitor could escalate toxicity, potentially hindering patient compliance and undermining the control of the metastatic lung carcinoma.

Another promising way to detect a *BRCA* reversion mutation is through circulating cell-free DNA (cfDNA). In a study conducted at a single institution using a clinically validated 73-gene cfDNA assay, which assesses single-nucleotide variants and insertion-deletion mutations (indels) in *BRCA1/2* and differentiates somatic/reversion from germline mutations with remarkable accuracy, the analysis extends beyond identifying germline and somatic *BRCA1/2* mutations. This cfDNA analysis methodology also enables the detection of reversion *BRCA1/2* mutations [14]. Among 828 individuals with advanced malignancies, including breast cancer, who underwent testing with the 73-gene cfDNA assay, 7.2% of the patients were found to carry one or more pathogenic *BRCA1/2* mutations. Notably, both somatic and germline variants were identified. Furthermore, in 21.4% of patients harboring germline *BRCA1/2* mutations, polyclonal reversion mutations were detected. These reversion mutations were most observed in individuals who had previously received a PARP inhibitor.

It is our assertion that individuals afflicted with metastatic lung adenocarcinoma should undergo thorough NGS procedures. These NGS assessments are indispensable for detecting infrequent yet exploitable mutations that can be effectively managed using tailored molecular-based therapies, thus augmenting the efficacy of disease control. Additionally, as the disease progresses, patients should pursue NGS analyses to unravel the intricate molecular mechanisms that underlie treatment resistance.

## Data Availability

Data will be made available under reasonable request.

## Abbreviations

cGy: Centigray
CNS: Central nervous system
NGS: Next-generation sequencing
PARP: Poly ADP-ribose polymerase
PET/CT: Positron emission tomography/computed tomography
SBRT: Stereotactic body radiation therapy
